# Telehealth Before and During the COVID-19 Pandemic: Analysis of Health Care Workers' Opinions

**DOI:** 10.2196/29519

**Published:** 2022-02-25

**Authors:** Pascal Nitiema

**Affiliations:** 1 Division of Management Information Systems Price College of Business University of Oklahoma Norman, OK United States

**Keywords:** telehealth, telemedicine, COVID-19, pandemic, physical examination, sentiment score, structural topic modeling, opinion, health care worker, social media, discussion

## Abstract

**Background:**

The COVID-19 pandemic and the lockdowns for controlling the spread of infection have led to a surge in telehealth adoption by many health care organizations. It is unclear how this pandemic has impacted health professionals’ view about telehealth. The analysis of textual data, such as comments posted on a discussion forum, can uncover information that may not be captured by a structured survey.

**Objective:**

This study aims to examine the opinions of health care workers about telehealth services during the time frame of March 2013-December 2020.

**Methods:**

Comments about telehealth posted by health care workers from at least 46 countries were collected from an online discussion forum dedicated to health professionals. The analysis included the computation of sentiment scores from the textual data and the use of structural topic modeling to identify the topics of discussions as well as the factors that may be associated with the prevalence of these topics.

**Results:**

The analysis of the comments revealed positive opinions about the perceived benefits of telehealth services before and during the pandemic, especially the ability to reach patients who cannot come to the health facility for diverse reasons. However, opinions about these benefits were less positive during the pandemic compared to the prepandemic period. Specific issues raised during the pandemic included technical difficulties encountered during telehealth sessions and the inability to perform certain care routines through telehealth platforms. Although comments on the quality of care provided through telehealth were associated with a negative sentiment score overall, the average score was less negative during the pandemic compared to the prepandemic period, signaling a shift in opinion about the quality of telehealth services. In addition, the analysis uncovered obstacles to the adoption of telehealth, including the absence of adequate legal dispositions for telehealth services and issues regarding the payment of these services by health insurance organizations.

**Conclusions:**

Enhancing the adoption of telehealth services beyond the pandemic requires addressing issues related to the quality of care, payment of services, and legal dispositions for delivering these services.

## Introduction

Telehealth services have been described by the World Health Organization as having a “great potential to address some of the challenges faced by both developed and developing countries in providing accessible, cost-effective, high-quality health care services” [1]. Multiple efforts have been undertaken across countries to increase the scope and reach of these services. Although the rate of adoption of telehealth by health care organizations in the United States has been steadily increasing, the percentage of hospitals using telehealth services is still relatively low [2,3]. Only 15% of physicians in the United States worked in health practices that used telehealth services in 2016 [3].

The COVID-19 pandemic and the lockdowns mandated by public authorities to reduce the spread of infection have led to a renewed interest from health care organizations in adopting telehealth platforms for some of their routine patient care operations. Event system theory posits that organizations are dynamic entities that can respond to events that are novel, critical (ie, the extent to which the event is deemed significant for the organization), and disruptive by altering their routine practices so that they can adapt to these events [4]. These unexpected events, because of their novel nature, usually find the organizations ill-prepared, causing the interruption of day-to-day operations and requiring changes to existing practices or the adoption of new ones [5]. Hence, it is not surprising that health care organizations have turned to telehealth services to accommodate the disruptions in patient care operations that were caused by the pandemic. Telehealth services allow long-distance patient care through telecommunication channels and thus do not require that patients and health care workers involved in the care process be physically in the same room. This feature made telehealth useful for adapting to the mandated lockdowns and the safe physical distancing measures advocated during the pandemic.

However, the successful implementation of changes in processes requires the adherence of organizational members. An unexpected event disrupts not only the routines of organizations but also the automatic cognitive processing employees use to perform their tasks [6]. Automatic cognitive processing, also called automatic information processing, is built through “repetitive training on the same task” and allows performing job-related tasks relatively swiftly and efficiently with reduced cognitive effort [7]. For instance, clinicians who have conducted physical examinations of hundreds or thousands of patients have developed heuristics and routinized practices for arriving at a diagnosis after detecting specific lung or heart sounds during auscultation. The disruption in automatic cognitive processing and the need to acquire new skills proper for the new circumstances created by a disruptive event impose undue burden on the employees and can lead to occupational stress and job dissatisfaction. However, the adoption of new practices can also be positively perceived and considered the rightful way for navigating the challenges created by the disruptive event.

The objective of this research is to examine the opinions of health care workers about telehealth services expressed before and during the COVID-19 pandemic. Comparing comments posted during the 2 time frames will illustrate the influence of the pandemic on the viewpoints of these health care workers on telehealth. The analysis of these opinions can shed light on the obstacles encountered by health care professionals using these services and help identify solutions to promote the adoption of these services.

## Methods

### Definition of Telehealth

The definitions of telehealth and telemedicine often vary across authors. In this manuscript, *telehealth* is defined as the umbrella term for a set of activities and services performed by health care professionals through telecommunication technologies to “support and promote long-distance clinical health care, patient and professional health-related education, public health and health administration” [8]. Hence, telehealth includes collaboration among health care workers discussing and sharing patients’ information through telecommunication channels, data collection and remote monitoring of patients’ health outcomes through digital wearables, and electronic transmission of prescriptions to pharmacists (e-prescribing). *Telemedicine*, a subset of telehealth, is strictly defined as the diagnosis and treatment of patients through telecommunication technologies. Nevertheless, the term *telehealth* will be used throughout the manuscript, except when analyzing or quoting health care workers’ comments, to encompass all health care operations performed through telecommunication technologies.

### Data Collection

The comments were collected from Medscape, an online platform that provides health care–related news to health professionals and hosts forums on health-related issues. According to the web analytics company similarweb, the online platform Medscape had more than 21 million visits per month during the summer and fall seasons of the year 2020. Most of the visits were from the United States (53%). The list of countries of the platform visitors included the United Kingdom (4%), Australia (4%), Canada (4%), India (3%), the Philippines (2%), and Brazil (2%). The keywords used to search for the comments were *telemedicine*, *telehealth*, *televisit*, *virtual visit*, *online visit*, and *video visit*. Available data on the characteristics of the commenters were collected as well, including the country of residence, occupation, and medical specialty when the commenter was a physician. Since the comments posted on the platform are publicly available and can be accessed by anyone, the institutional review board of the University of Oklahoma, USA, determined that this investigation did not require ethics committee approval.

### Data Analysis

The sentiment scores of the collected comments were computed using the R package *sentiment*, which considers valence shifters (eg, happy vs not happy) and (de-)amplifying words (eg, very worried vs slightly worried) [9]. A mixed-effect model with random intercepts was fitted to assess the association between sentiment scores and the following independent variables: residence of the commenter (US vs non-US), occupation (physician, nurse, health administrator, other) of the commenter, and the year in which the comment was made. The restricted maximum likelihood method was used to estimate the model parameters, and the covariance pattern of the model was set to unstructured.

**Figure 1:**
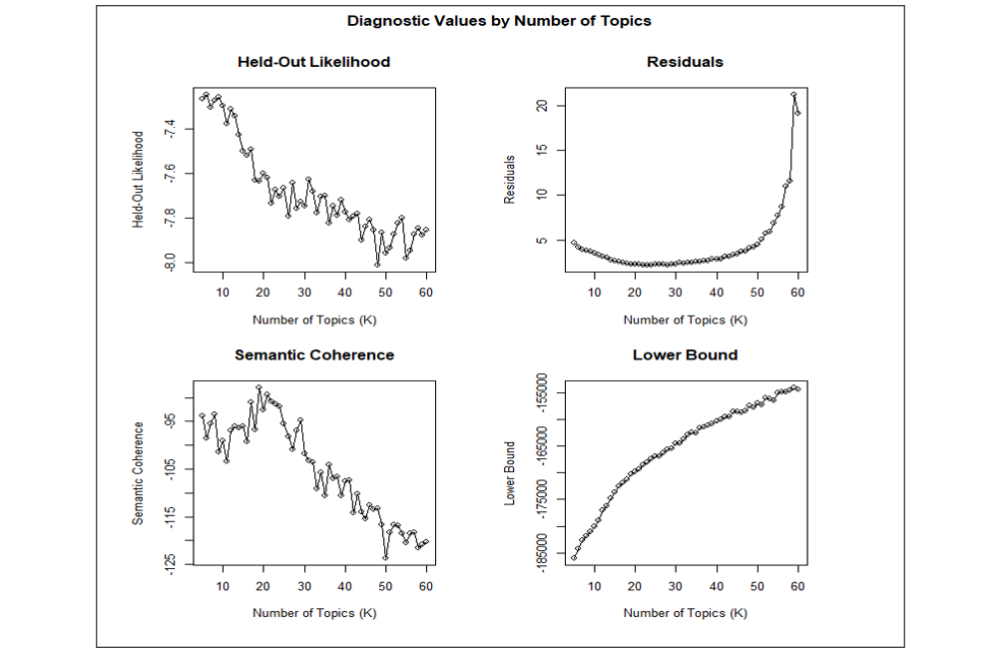
Diagnostic values by number of topics.

Examining the prevalence of discussion topics across time frames and commenters’ characteristics can shed light on the opinions or concerns about telehealth that are specific to these time frames and these individual characteristics. Structural topic modeling (STM) was used to identify the topics discussed in the comments. STM allows the analyst to incorporate information about the characteristics (metadata) and content of the documents into the modeling process [10]. The following metadata were used as independent variables to estimate the topic prevalence in the comments: the residence (US vs non-US) and occupation (physician, nurse, health administrator, other) of the commenter, the time frame in which the comment was posted (before vs during the pandemic), and the average sentiment score of the comment. The date of March 11, 2020, the day the World Health Organization declared the COVID-19 outbreak a pandemic, was used as the cut-off date for defining the time period [11]. First, the texts of the comments were processed by removing stop words using the System for the Mechanical Analysis and Retrieval of Text (SMART) stop word list. Then, nouns in plural were converted to their singular forms. No stemming was performed. The appropriate number of topics for the corpus of comments was determined to be between 10 and 25 by examining the values for the held-out likelihood, semantic coherence, residuals, and lower bound [12]. See Figure 1. The appropriate number of topics to extract was determined by selecting the model that yielded both the highest semantic coherence and the highest exclusivity values. The number of topics found to have optimal exclusivity was 13, and thus, 13 topics were selected for modeling. Spectral initialization was used for the topic modeling, which converged after 500 iterations, and 4 single-word comments were dropped during the modeling process. The final model consisted of 13 topics, 910 documents, and a 3252-word dictionary. The correlations among the identified topics were computed with the maximum a posteriori estimates of the topic proportions. A threshold of *r*=0.15 for the correlation was set to determine whether 2 topics were correlated; that is, 2 topics were considered uncorrelated if their pairwise correlation was less than 0.15. STM was performed using the R package *stm* [12].

## Results

### Characteristics of Health Care Workers

A total of 914 comments made by 705 health care workers were collected from the forums. The dates of the comments spanned from the year 2013 through 2020, and the commenters were from at least 46 countries in 6 continents (Africa, Asia, Australia, Europe, North America, and South America). The majority of commenters were physicians (n=468, 66.4%) and lived in the United States (n=561, 79.6%). The characteristics of the 705 health care workers are presented in Table 1. Of the 914 comments, 583 (63.8%) were posted from the year 2013 up to the day the COVID-19 outbreak was declared a pandemic by the World Health Organization on March 11, 2020, and 331 (36.2%) were posted during the pandemic (ie, mid-March 2020-December 2020). The lengths of the comments ranged from 1 word to 630 words (mean=81.9, median=60.5, SD 80.4).

**Table 1:** Characteristics of health care workers who commented on telehealth.

Characteristics		Individuals’ frequency, n (%)
**Region (N=705)**		
	Northern America	561 (79.6)
	Asia	37 (5.2)
	Europe	23 (3.3)
	Africa	19 (2.7)
	Central or South America	12 (1.7)
	Australia or New Zealand	10 (1.4)
	Unknown	43 (6.1)
**Occupation/position (N=705)**		
	Physicians	468 (66.4)
	Nurses	89 (12.6)
	Health administration professionals	42 (6.0)
	Other health occupation	105 (14.9)
	Unknown	1 (0.1)
**Physicians’ medical specialties (N=468)**		
	Family medicine/general practice	106 (22.7)
	Clinical medicine specialties	254 (54.3)
	Surgery and surgical specialties	45 (9.6)
	Mental health	45 (9.6)
	Other specialty	5 (1.1)

### Sentiment Scores of Comments

**Figure 2:**
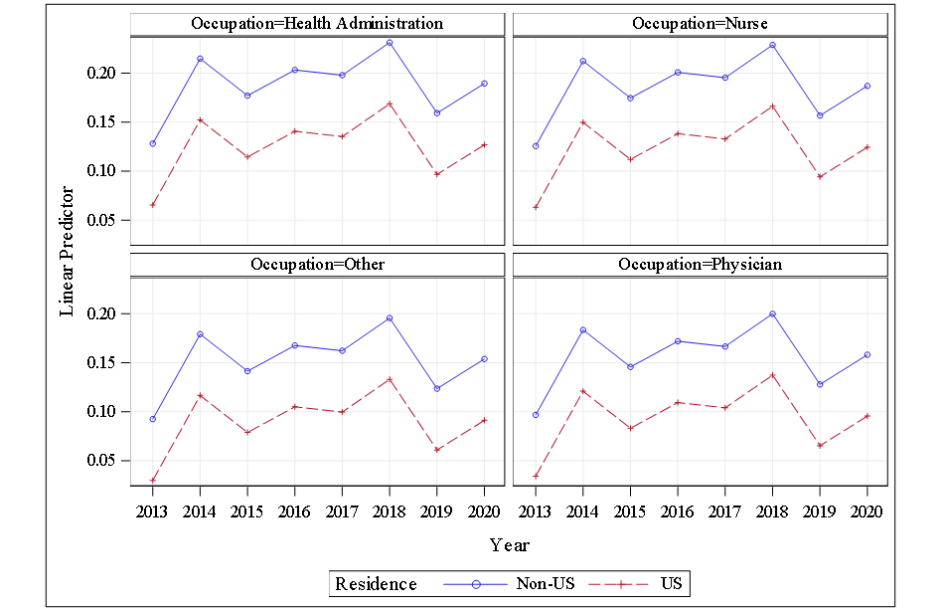
Average sentiment score per sentence of comments about telehealth by occupation and by residence.

The mixed-effect model with the comment sentiment score as the dependent variable showed that, in general, comments made by health care workers living in the United States were less positive compared to their counterparts residing outside the United States. There were no statistically significant differences in average sentiment scores among occupations (physician, nurse, health administration professional, other) or across years in which comments were posted (2013-2020). Hence, overall, the sentiment scores of comments posted during the pandemic were comparable to those of comments made before the pandemic. See Table 2, Textbox 1, and Figure 2.

**Table 2:** Fixed-effect estimates of independent variables on sentiment scores of comments about telehealth services.

Independent variable	Coefficient estimate (95% CI)	*P* value
Residence: non-US vs US	0.06 (0.02-0.10)	.003^a^
Occupation: nurse vs physician	0.03 (–0.02-0.08)	.23
Occupation: health administration vs physician	0.03 (–0.04-0.10)	.38
Occupation: other vs physician	0 (–0.05-0.04)	.85
Year 2013 vs 2020	–0.06 (–0.18-0.06)	.31
Year 2014 vs 2020	0.03 (–0.01-0.07)	.21
Year 2015 vs 2020	–0.01 (–0.06-0.04)	.62
Year 2016 vs 2020	0.01 (–0.03-0.06)	.58
Year 2017 vs 2020	0.01 (–0.06-0.08)	.81
Year 2018 vs 2020	0.04 (–0.13-0.21)	.63
Year 2019 vs 2020	–0.03 (–0.09-0.02)	.28

^a^*P*<.01.

**Model statistics.**

Null model likelihood ratio:

χ^2^=9.80, *df*=1, *P*=.002

Random effects:

z=2.83, *P*=.002^a^

### Topics of Discussions

STM identified 13 topics presented in Table 3. The relationships between the prevalence of the identified topics and the characteristics of the comments or commenters are presented in Table 4. There was no correlation among the 13 identified topics, that is, all pairwise correlations among topics were less than 0.15. The 3 most discussed topics were virtual visits (topic 1; 10.2% of comments), which are live encounters between a patient and a health care worker through a telecommunication channel (video, telephone, etc); the potential benefits of telehealth (topic 2; 10%); and criticisms of telehealth (topic 3; 9.4%).

**Table 3:** Identified topics from comments (N=914) about telehealth made by health care workers.

Topic	Percentage of Topic in Corpus	Top 10 highest-probability words	Quotes
Topic 1: virtual visits	10.2	visit, patient, virtual, office, time, doctor, video, work	*My physician's office is seeing patients using virtual visits, when possible, through May.* *Anyone who thinks a virtual office visit provides the same quality of care as an in-person office visit is seriously deluded. Yet, there clearly is a benefit from this technology in terms of accessibility, and I have used it myself to help people who are unable to visit my office because of debility.*
Topic 2: benefits of telehealth	10.0	care, patient, technology, good, provider, great, telemedicine, service	*Telemedicine is good if a patient unable to go to their doctors because of many reasons.* *You are missing the point of telemedicine. It is designed to provide quicker service, [lessen the] burden of travel expenses for the patient, and increase the point of access of care to allow observation and improved treatment plan adherence. Overall, if this is done responsibly, more patients are provided better service, [and] compliance recidivism decreases.*
Topic 3: criticisms of telehealth	9.4	telemedicine, patient, exam, physical, care, antibiotic, medicine, physician	*I would opt for physical examination of the physical body, rather than virtual examination via telemedicine, every time.* *There are already studies that show that clinicians using telemedicine are more likely to (over)prescribe antibiotics.*
Topic 4: insurance payment for telehealth services	8.8	patient, phone, care, time, pay, make, insurance, practice	*I've found 90% of cases can be managed over [the] phone or videoconference. If they offer similar reimbursement from insurance, this may be the way to practice in the future.* *The government allows doctors to practice telemedicine across state lines but won't allow insurance companies to bid and provide across state lines?*
Topic 5: telehealth over the phone	8.0	telehealth, physician, health, work, patient, call, medicine, clinic	*Vomiting and diarrhea on Saturday morning. Call primary care physician (35-year history as doctor/patient) for prescription. Answering service: “Sorry, can't prescribe by phone. Go to convenient care.” “Can't. Too sick.” (Silence.) “Hello, telehealth.”* *We do a follow-up telehealth call to reach out to review labs, etc, with the patients about 2 weeks later. Great for our shut-in patients. They are willing to pay the extra fees associated for the convenience of this service.*
Topic 6: legal dispositions for telehealth	7.6	state, medicine, license, practice, board, health care, patient, telemedicine	*The current system requires that doctors who practice telemedicine need a license in each state where potential telemedicine patients are. Consequently, a telemedicine doctor in Utah [who] treats patients in Wyoming needs both state licenses.* *Can a physician licensed in a foreign country provide telehealth services to patients in said country while being on US soil?*
Topic 7: practice of medicine in the era of telehealth	7.4	medicine, patient, doctor, clinical, care, practice, physician, service	*Let the primary physician do the family practice and refer the cases to the respective specialties. Otherwise, patients will become “Amish.”* *The inhibition of the nonavailability of direct physical assessments is the major legal hurdle for legalizing the authority of prescribing medicines to telemedicine physicians. But dear, this needs to be seen as follows: To overcome these inhibitions, a doctor who is competent in subject could assess the complaints, in the absence of physical findings, by virtue of [their] experience in assessing the clinical value of complaints of telepatients, who currently use teleservices for [a] second opinion.*
Topic 8: moving consultations to the patients’ home	7.4	people, patient, health care, work, year, home, telemedicine, health	*Allowing the patient to be seen in the familiar comfort of their home seems to provide a more optimal environment for cooperation.* *Huge opportunity for technology to improve health care delivery. The fact that 90% of people have a smartphone opens up endless possibilities to improve how we interact with and support patients. Despite the long-standing emphasis on traditional office visits, the effectiveness of these encounters is not reassuring.*
Topic 9: impact on patient-physician relationship	7.1	physician, patient, primary care, relationship, medicine, system, health care, doctor	*This certainly creates distance between the patient and physician, decreasing the interpersonal relationship and the comfort of human touch, which is tremendously important in [a] bedside manner.* *Believe it or not, there is no perfect technology that will replace the personal relationship of a doctor with the patient.*
Topic 10: physical examination in the era of telehealth	6.4	patient, exam, care, hand, medicine, system, test, doctor	*The basic tenet of medicine is laying your hands on the patient and examining what's wrong. Telemedicine is junk medicine except for maybe psychiatric consultations. It will drive up costs gradually, with doctors ordering unnecessary diagnostic tests and reviewing results online and treating results instead of the patient. Instead of unifying care and the concept of a medical home, it will splinter care.* *In-office visits will soon become archaic remnants of a past rooted in blindly following the old ways. Why would a patient be subject to the time, inconvenience, and danger by subjecting themselves to an in-office visit? And likewise, why would the health care system subject itself to the expense of maintaining staff and facilities necessary for in-office visits? Use of facilities should be restricted to hands-on procedures and assessments only or those requiring specialized equipment.*
Topic 11: impact of telehealth on care quality	6.2	doctor, patient, time, medicine, bad, family, day, registered nurse (RN)	*The issue of bad medicine is rampant; a consumer requests something, and the prescribing doctor writes for it to make them happy. Telemedicine just makes this problem worse.* *Medical boards will become involved in it at some point when a bad outcome occurs and family members get upset over that bad outcome. There will have to be some sort of informed consent that the patient will have to accept the risks of no physical assessment and perhaps an incorrect diagnosis.*
Topic 12: issues related to telehealth patient appointment and follow-up	5.9	patient, phone, call, appointment, follow, issue, physician, telemedicine	*It is also burdensome to contact any health care provider via a telephone call. So, what are patients and health care providers without access to the necessary technology supposed to do regarding annual follow-up appointments and other medical visits requiring actual physical re-examinations.* *Physicians should certainly be reimbursed for patient care, including videoconferencing, and follow-up phone calls.*
Topic 13: advantages of telehealth for remote and rural areas	5.6	telemedicine, patient, rural, clinic, medicine, hospital, care, remote	*Definitely virtual doctors are a boon to remote, rural areas where [a] doctor is a rare commodity.* *This works great for people living in rural areas, with limited access to health care services.*

**Table 4:** Coefficient estimates of independent variables across the 13 identified topics.

Independent variables	Topic 1: virtual visits, β; *P* value	Topic 2: benefits of telehealth, β; *P* value	Topic 3: criticisms of telehealth, β; *P* value	Topic 4: payment of services, β; *P* value	Topic 5: telehealth over the phone, β; *P* value	Topic 6: legal dispositions for telehealth, β; *P* value	Topic 7: practice of medicine, β; *P* value	Topic 8: Moving consultations into patients’ home, β; *P* value	Topic 9: impact on patient-physician relationship, β; *P* value	Topic 10: impact on physical examination, β; *P* value	Topic 11: impact on quality of care, β; *P* value	Topic 12: patient appointment and follow-up, β; *P* value	Topic 13: telehealth for remote and rural areas, β; *P* value
During vs before pandemic	0.05; .002^a^	–0.03; .04^b^	0.10; .04^b^	0.02; .31	0.04; .01^a^	–0.01; .45	–0.06; <.001^a^	0.07; <.001^a^	–0.02; .09	–0.04; .02^b^	–0.04; .01^a^	0.01; .48	0.08; <.001^a^
Sentiment score	0.04; .32	0.35; <.001^a^	–0.14; <.001^a^	0; .97	–0.03; .38	0; .98	0.05; .25	0.04; .25	–0.08; .03^b^	–0.06; .12	–0.12; <.001^a^	–0.03; .19	–0.02; .56
Pandemic^b^ sentiment score	0.06; .38	–0.19; .004^a^	0.05; .42	0.05; .48	–0.05; .43	0; .97	0; .99	0.02; .79	–0.02; .80	–0.03; .61	0.12; .04^b^	–0.01; .89	0; .98
Residence: non-US vs US	–0.02; .29	0.01; .46	–0.03; .19	–0.06; .004^a^	–0.01; .63	–0.06; .002^a^	0.15; <.001^a^	0.02; .28	–0.01; .59	–0.04; .04^b^	–0.01; .63	0.02; .19	0.01; .67
Nurse vs physician	–0.02; .22	0.03; .09	–0.06; .002^a^	–0.04; .05	0.03; .08	–0.03; .20	–0.04; .05	0.04; .05	–0.01; .47	0.04; .05	0; .80	0.05; .004^a^	0; .84
Health administrator vs physician	–0.01; .71	0.03; .25	–0.06; .03^b^	0; .88	–0.02; .49	–0.03; .31	0.02; .62	0.04; .16	–0.02; .46	–0.03; .31	–0.03; .31	0.08; <.001^a^	0.02; .41
Other vs physician	0.03; .09	0.04; .04^b^	–0.01; .54	0; .98	0; .85	–0.02; .24	0.06; .01^a^	0.01; .70	–0.04; .03^b^	–0.04; .04^b^	0; .99	0; .86	–0.03; .11

^a^*P*<.01.

^b^*P*<.05.

The frequently mentioned benefits (topic 2) included the possibility telehealth offers to reach patients who cannot come to the health care facility for some reasons (living with a disability, residing in a remote area, etc), the decrease in the transportation time and cost for the patient, and the improvement in patients’ access to care and to medical specialists. Some of these benefits were emphasized during the pandemic:

[The] majority of my patients have been so glad that we were able to continue with therapy during the pandemic. Some will be happy to get back to face-to-face therapy, but most have seen no change in the sessions.

Although the topic on the benefits of telehealth (topic 2) was associated with a positive sentiment score overall, the sentiment score for that topic was less positive during the pandemic compared to its prepandemic value. A deeper examination of the comments revealed that this decrease in the sentiment score may have stemmed from the changes in routine care processes due to pandemic lockdowns and from the technical difficulties encountered by health care workers who had acknowledged the value of telehealth services:

Change is difficult, but telehealth works great for the provider as well as the patient.

Virtual visits for pediatrics will be quite difficult. Limited virtual consultation may be possible, particularly with visually obvious abnormalities that can be followed visually. I cannot visualize evaluations of children with epilepsy, asthma, autism spectrum, cerebral palsy . . . being done by virtual video visits.

Sure, telemedicine during a global pandemic to enable social distancing is to be expected, but it is far from a panacea . . . No one also talks about technological limitations . . . I didn't go into health care to troubleshoot people's internet connections. I'd say 50% of my telemedicine encounters have some sort of technical issue that we spend easily the first 10 minutes dealing with.

Criticisms of telehealth (topic 3) included the impracticality to perform a physical examination of the patient, which may lead to misdiagnoses, and the overprescription of certain medications by health care providers who used telemedicine (eg, antibiotics). Health care workers who raised these criticisms opined that telehealth devalues “the art and science” of medicine:

Telemedicine makes a mockery of the art of medicine.

Say goodbye to the thorough, hands-on assessment and the art of medicine.

Some commenters argued that the resistance to telehealth from some health care professionals may be due the noninclusion of telehealth services into the curricula of training institutions:

The problem with telemedicine is that it flies in the face of how many of us were trained. We were constantly told to examine the patient and pay less attention to labs and radiology.

Worst of all, very few have really been trained on telemedicine, and how to handle its limitations and pitfalls.

Other workers stated that adequate training may help decrease the observed resistance to telehealth services and lead to improved quality of these services:

With proper training and some careful forethought, telehealth can be done well for those with serious illness.

With appropriate training regarding the manner in which this kind of therapeutic contact is to be carried out, videoconferencing can be a valuable conduit for the delivery of clinical treatment or educational services.

Other topics raised by commenters included the payment of telehealth services by insurance companies (topic 4; 8.8%) and the legal dispositions necessary for the practice of telehealth (topic 6; 7.6%), especially an interstate medical licence for physicians who practice in the United States. Comments on the lack of proper legal dispositions for telehealth were not specific to health care workers from the United States, however. For instance, a physician from Brazil noted that

Here where I am (Brazil), there are a series of legal requirements that still discourage (or scare) a professional using video calls. Psychologists are more advanced on this topic, but doctors are not yet. The COVID-19 pandemic is accelerating the discussion, but there are still many barriers and doubts.

Virtual visits (topic 1), criticisms of telehealth (topic 3), telehealth services (topic 5), moving consultations to patients’ homes (topic 8), and the advantages of telehealth for remote areas (topic 13) were more frequently discussed during the COVID-19 pandemic compared to the prepandemic period. In contrast, the benefits of telehealth (topic 2), the practice of medicine (topic 7), physical examination in the era of telehealth (topic 10), and the impact of telehealth on care quality (topic 11) were less frequently discussed during the pandemic compared to before the pandemic. Compared to their counterparts living in other countries, health care workers in the United States discussed insurance payment of telehealth services (topic 4), the legal dispositions for telehealth (topic 6), and physical examination in the era of telehealth (topic 10) more frequently. Non-US health care workers, instead, discussed the practice of medicine in the era of telehealth (topic 7) more frequently than their US counterparts. In general, criticisms of telehealth (topic 2) were less frequently discussed by nurses and health administrators compared to physicians. However, nurses and health administrators discussed issues related to telehealth patient appointments and follow-ups (topic 12) more than physicians.

Although the topic of the impact of telehealth on care quality (topic 11) was associated with a negative sentiment score overall, the score was less negative during the pandemic compared to the prepandemic period, signaling a shift in opinion. Such shift can be seen in comments such as the following posted during the pandemic:

Having previously been against telemedicine, now I see a lot of its positive qualities, specifically for the elderly and for the young, however, too.

The telemedicine services were underutilized. The new pandemic shows the importance of telemedicine. The wider use of smartphones is also helpful in this direction.

However, the adoption of telehealth services by some health care workers may not outlast the pandemic, as illustrated by this statement posted by a physician:

The telemedicine visit was important for the first 3 months of the pandemic. However, as soon as we were able to see patients in the office, then that became my preferred method to evaluate patients. I agree that [a] lack of the physical exam was the major reason to encourage in-office visits, even with stable, chronic disease patients.

## Discussion

### Principal Findings

The objective of this research was to examine the opinions of health care workers about telehealth services and to explore the impact the COVID-19 pandemic may have had on these opinions. The analysis showed that the COVID-19 pandemic did not significantly alter the opinions about telehealth services expressed by health care workers on the discussion forum of the study. Although some topics were more (eg, topic 1: virtual visits) or less (eg, topic 7: practice of medicine) frequently discussed during the pandemic compared to the prepandemic period, the same issues about telehealth services were raised during both time frames. Furthermore, the topic of telehealth benefits (topic 2) was associated with a less positive sentiment during the pandemic compared to before the pandemic. However, the topic of the quality of care of telehealth services (topic 11) had a higher positive sentiment score during the pandemic compared to the prepandemic time frame. This finding may be reflecting the opinion among some health care workers that telehealth can enhance the quality of care only in unusual situations when an in-person encounter between patient and clinician is not feasible, as was the case during the pandemic.

### Differences in Comment Sentiment Scores

The sentiment scores of comments posted by health care professionals living in the United States were less positive than those of opinions expressed by their counterparts living in other countries. The lack of additional information about the characteristics of the commenters (eg, sociodemographics, computer knowledge) did not allow the assessment of whether this association between location of residence and sentiment scores was due to differences in sociodemographics or in the knowledge of information technology (IT) between commenters from the United States and their counterparts from other countries. Indeed, previous studies have reported that the attitude toward technology may differ by age, occupational seniority, and level of training in IT [13,14]. It is worth emphasizing that the general trends in sentiment scores do not necessary reflect sentiments about telehealth per se, as demonstrated by the multiple topics and the associated sentiment scores uncovered by the analysis. Rather, these general trends show the aggregate sentiment of the concerns or expectations elicited by discussions of telehealth.

### Discussion Topics Associated With Negative Sentiment Scores

Although the pandemic may have offered the opportunity to some health care workers to change their opinions about telehealth services, criticisms of these services were still relatively prevalent among the commenters during the pandemic. Reports on the drawbacks of telehealth, such as the overprescription of antibiotics in direct-to-consumer (DTC) telehealth visits, were frequently mentioned by health care workers who had an unfavorable opinion of telehealth services [15]. DTC telehealth providers offer virtual consultations with health care workers to customers or patients through applications installed on the IT devices of these patients. Patients enter their symptoms or motives of consultation in the application and are then assigned to a health care worker in the network of the DTC telehealth company. The interaction between the patient and the health professional is entirely virtual, and the latter may prescribe a treatment at the end of the encounter. DTC telehealth has often been associated with reduced care quality [16]. It should be mentioned that other types of telehealth services that involve some degree of clinical or paraclinical examination have yielded good quality care [17-19]. Another disadvantage of telehealth that was frequently mentioned before and during the pandemic was the inability to perform a physical examination. Conducting physical examinations has been reported to be perceived by health care workers as part of their identity [20]. Hence, telehealth platforms that do not offer the possibility to perform a physical examination are perceived to threaten that identity. Statements such as “telemedicine makes a mockery of the art of medicine” can be interpreted through the lens of the perceived threat to this occupational identity. It is worth noting that some solutions exist to address the issue of physical exam during telehealth sessions. These solutions include facilitated virtual visits, which require the patient to be examined in a designated facility (originating site), usually by a health care professional (facilitator), during the virtual visit. Information about the physical exam is then transmitted by the facilitator to the patient health provider participating in this telehealth session. Another solution for addressing the issue of physical examination is the use of diagnostic tools (eg, digital stethoscope) that can transmit data from the physical examination remotely [21].

### Promoting the Adoption of Telehealth Services

Researchers have shown that the technology acceptance model [22], which posits that the individuals’ perceptions of the technology’s usefulness and ease of use influence their acceptance of that technology, was an adequate framework for predicting health care professionals’ intention to use telehealth services [23,24]. Hence, promoting the usefulness of telehealth services and implementing systems that are perceived as easy to use can boost the adoption of telehealth by health professionals. In this study, topics related to the ease of use of telehealth included not only its limitation for conducting a physical examination (topic 10) but also the lack of appropriate legal and service reimbursement dispositions for its adoption (topics 4 and 6). Hence, enhancing telehealth adoption requires addressing these barriers to its usage. Topics related to the perceived usefulness of telehealth included discussions of its benefits (topic 2) and the advantages it offers for the care of patients living in remote or rural areas (topic 13). However, the promotion of the usefulness of telehealth will need to address the perceived drawbacks of its adoption, namely its reported or perceived negative impact on the quality of care (topic 11) and the practice of medicine (topic 3).

Serrano and Karahanna [25] proposed that a successful session of a telehealth visit requires 3 ingredients: the ability of the patient to relay the relevant information through the technology, the competence of the clinician in eliciting from the patient the appropriate information needed to perform the clinical service, and the capacity of the technology to transmit and present the relevant information to both the clinician and the patient. Health professionals’ frustrations from telehealth may come from the patients’ lack of knowledge on how to use the technology to convey information relevant to their condition. A physician wrote,

I put myself in the place of a patient who only knows Skype and who, from one moment to the next, to talk to his doctor, will need to use a platform he has never seen . . . it must be very embarrassing.

Hence, educating patients on how to use a telehealth platform should be among the measures implemented to improve the quality of telehealth services. Health care professionals providing telehealth services should be trained as well, not only on how to navigate the technological features of the platform, but also on how to foster a virtual environment that promotes a good patient-clinician relationship that can help obtain relevant clinical information from the patient. As demonstrated by Serrano and Karahanna [25], a clinician task-specific capability (eg, competency in patient history enquiry) can compensate for the limitations of technology in performing certain tasks. In addition, the technology should fit the clinical task to be performed. Many of the criticisms against telehealth from clinicians included the difficulty or impossibility to obtain certain information through the platform. Under the task-technology-fit framework, the platform used for the telehealth should include features that enhance image or sound quality so that the virtual patient-clinician encounter can be as close as possible to an in-person interaction [25]. Additional technological requirements include a secure and adequate internet connection and easy-to-use software [21]. There are platforms that allow performing a certain number of physical examinations (called provider access software), but their capabilities are still limited [21]. Finally, a technological support team should be available when the system malfunctions.

Factors that may improve health care workers’ perceptions about telehealth include involving them in the design and implementation of the organization’s telehealth platforms [26], having opinion leaders express their support for these services and making resources and technical support available [27], and training adequately workers in utilizing these services [28].

The absence of adequate legal dispositions and the differences in telehealth service payment policies by health insurance companies, as noted by some commenters, can hamper the adoption of telehealth [29]. Adler-Milstein et al [30] reported that in the United States, the promotion of private payer reimbursement policies for telehealth services enhances telehealth adoption, while the requirement of an out-of-state licensure for telehealth decreases its adoption. Hence, wider structural changes beyond individual health care organizations are needed as well to foster the adoption of telehealth services.

### Limitations

The study had some limitations. First, the sample of health care workers who posted their comments on telehealth are not necessarily representative of the population of health care professionals working in the United States or in other countries. In addition, the relatively small number of comments collected may not have been exhaustive enough to identify other prevalent viewpoints about telehealth. Finally, there is no available method for determining the exact number of topics to be extracted from a corpus. Methods available are all approximate and use different criteria for selecting the number of topics to extract. Thus, it is possible that methods different from the ones used in this paper (semantic coherence, held-out likelihood, and exclusivity) may have yielded a different value for the number of topics to be extracted. Despite these limitations, the analysis of the collected comments provided a valuable insight into the opinions of health care workers about telehealth services over a period of 8 years (2013-2020).

### Conclusion

The COVID-19 pandemic and the lockdowns mandated by public officials to control the spread of infection have fostered an interest in expanding telehealth services among health care organizations. However, hurdles to the widespread adoption of these services still remain, including some health care workers’ resistance to telehealth, insufficient or inadequate legal dispositions for providing these services, and a lack of coverage of these services by insurance companies. Promoting changes in the unfavorable attitude toward telehealth among health care professionals may require the promotion of evidence-based literature that demonstrates high satisfaction of both patients and clinicians about telehealth. Furthermore, including telehealth service training into the curricula of health care professional training institutions can help prepare these workers perform these services.

## Abbreviations

DTC: direct to consumer
IT: information technology
STM: structural topic modeling
